# Report of the multistakeholder drug development round table meeting of the World Duchenne Organization focusing on challenges for clinical development of therapies

**DOI:** 10.1177/22143602251399511

**Published:** 2025-12-04

**Authors:** Annemieke Aartsma-Rus

**Affiliations:** Department of Human Genetics, Leiden University Medical Center, Leiden, the Netherlands

**Keywords:** Duchenne muscular dystrophy, clinical trials, trial design, gene therapy

## Abstract

Duchenne muscular dystrophy (DMD) is a muscle wasting disease where patients lose muscle tissue and function. The disease is caused by pathogenic variants that abolish the production of functional dystrophin protein. There are many therapeutic approaches in clinical development for DMD patients, but so far showing clinical benefit in trials has proven challenging. On May 6, 2025, the World Duchenne Organization convened a multistakeholder drug development round table meeting to discuss pertinent aspects of drug development in the DMD field: trial design, the impact of variable doses and regimen of glucocorticoids on disease trajectory, gene therapy and the use of real world evidence. Herein the most important discussion points, realizations and recommendations are summarized. As current therapeutic approaches for DMD patients aim to slow down disease progression, measuring benefit will likely be challenging. The trial design should consider the mechanism of action of the therapeutic approach, the expected therapeutic effect, and the exposure to glucocorticoids could be considered as a stratification factor (regimen and when glucocorticoids were initiated). For gene therapy there are many uncertainties yet, and in hindsight trials were not all properly designed. Looking forward, additional data needs to be collected to assess the therapeutic effect and its longevity. Finally, real world data can only be used if it has sufficient quantity and quality. This will require global alignment and collaboration.

## Context

Duchenne muscular dystrophy (DMD) is a severe, progressive muscle wasting disorder, caused by pathogenic variants that prevent the production of functional dystrophin.^1^ Muscle damage is already present at birth, but the first clinical symptoms are often not recognized until around 3–4 years of age. These include gait abnormalities, frequent falls and difficulties climbing stairs. All patients develop dilated cardiomyopathy. Cognitive problems exist in 30% of patients, as well as behaviorial problems. Learning difficulties and speech delay are reported frequently.

The disease is caused by pathogenic variants in the *DMD* gene that prevent the production of dystrophin proteins in the skeletal muscle, heart and brain. The average age at which a genetic diagnosis is made is 4–5 years, often after a diagnostic odyssey.^2^

Once the diagnosis is made, symptoms management is provided as multidisciplinary care, which includes the use of glucocorticoids.^3–5^ High dose, daily glucocorticoid use (0.75 mg/kg/day prednisone; 0.9 mg/kg/day deflazacort; 6 mg/kg/day vamorolone) improves motor outcome performance and slows disease progression, but is accompanied by variable side effects including increased appetite, behavioral changes, growth stunting, puberty delay and osteoporosis.^6^ To manage these, the doses or regimen of glucocorticoids are often adjusted below standard of care recommendations or patients use on/off regimen.

Even with optimal care, the disease progresses and patients lose ambulation in the early teens, need assisted ventilation around the age of 20 and die due to heart or respiratory failure in the 2^nd^ – 4^th^ decade of life.^1^

## Current state of the art of therapy development

The dystrophin gene and protein were identified in 1986 and 1987^7^ and since then, approaches to restore the missing protein, or targeting pathology have been in development, first preclinically but in the last decades also clinically.^1^ Furthermore, several therapeutic approaches have received regulatory approval recently (Table 1). For two (givinostat and vamorolone) this was after clinical trials showed clinical benefit (see Table 2 for generally used outcome measures in the DMD field). Givinostat is an inhibitor of histone deacetylases (HDACs). These enzymes are overactive in DMD patients and this exacerbates the pathology by reduced dampening of the inflammatory response, increased formation of fat and fibrotic tissues and reduced regeneration. As such, inhibition of HDAC enzyme activity was speculated to be therapeutic as it would reduce pathology and improve regeneration in skeletal muscle. In a clinical trial in DMD patients, the velocity in the 4-stair-climb test was significantly better in givinostat treated than placebo treated DMD patients after 18 months of treatment.^8^

**Table 1.** Duchenne muscular dystrophy treatments with regulatory approval.

Therapy	Approved by	Type of approval (most recent)	Comments
Ataluren (Translarna)	EMA (2014–2025)	Conditional approval for ambulant patients 2 year and older (until 2025)	Two confirmatory placebo controlled trials failed to meet their primary endpoint (6MWD, 48 and 72 week studies); Conditional marketing authorisation was not renewed in 2025.
Eteplirsen (Exondys51)	FDA (2016)	Accelerated approval	Approval based on increased dystrophin expression, confirmatory studies to assess impact on disease progression ongoing
Golodirsen (Vyondys53)	FDA (2019)	Accelerated approval	Approval based on increased dystrophin expression, confirmatory studies to assess impact on disease progression ongoing
Viltolarsen (Viltepso)	FDA (2020) JMHWL (2020)	Accelerated approval	Approval based on increased dystrophin expression, confirmatory trial missed its primary endpoint (rise from supine after 48 weeks of treatment)
Casimersen (Amondys45)	FDA (2021)	Accelerated approval	Approval based on increased dystrophin expression, confirmatory studies to assess impact on disease progression ongoing
Delandistrogene Moxeparpovec (Elevidys)	FDA (2023)	Full approval for ambulant patients, accelerated approval for non ambulant patients (since 2024)	Accelerated approval for 4–5 year old patients was given in 2023, pending confirmatory trials, which failed to meet the primary endpoint (NSAA 48 weeks post treatment); Still full approval was given in 2024 for ambulant patients 4 years and older. CHMP did not approve Elevidys for 3–7 year old ambulatory patients.
Vamorolone	FDA (2023) EMA (2023)	Full approval for patients 2 years and older (FDA) and patients 4 years and older (EMA)	Based on a study comparing vamorolone with placebo showing similar functionality on the primary endpoint (time to rise from supine after 24 weeks of treatment) full approval was given
Givinostat	FDA (2024) EMA (2025)	Full approval for patients 6 years and older (FDA) Conditional approval for ambulatory patients 6 years and older on steroids (EMA)	Primary endpoint (4 stair climb velocity) was met in an 72 weeks placebo-controlled trial

EMA: European Medicines Agency; FDA: Food and Drug Administration, USA; JMHWL: Japanese Ministry of Health, Welfare and Labor; NSAA: North Star Ambulatory Assessment; 6MWD distance walked in 6 min in the 6 min walk test

**Table 2.** Outcome measures commonly used in DMD clinical trials.

Name	Context of use	Description
North star ambulatory assessment scale (NSAA)	Ambulatory DMD patients aged 4 and older	17 point item scale where patients score 2 (can perform), 1 (can perform independently with adaptations), 0 cannot perform
6 min walk test (6MWT)	Ambulatory DMD patients	Patients walk at maximum speed for 6 min without running on a flat surface and the distance walked (6MWD) is recorded; this test was developed as an outcome measure in the cardiorespiratory field
Performance upper limb (PUL)	Ambulatory and non-ambulatory DMD patients	22 item scale with focus on shoulder, elbow and distal domain function where patients score 2 (can perform), 1 (can perform independently with adaptations) or 0 (cannot perform)
Rise from supine)	Ambulatory DMD patients	The time it takes to rise from a supine position is measured.
Four stair climb (4SC)	Ambulatory DMD patients	The time it takes to climb four stairs is measured, usually depicted as the velocity
95^th^ percentile stride velocity (SV95C)	Ambulatory DMD patients	The average velocity of the 5% of quickest strides measured by an activity monitor over the period of at least active 100 h.

Vamorolone is a dissociative glucocorticoid developed as an alternative to prednisone and deflazacort. Vamorolone is expected to result in less side effects as it is an antagonist for the mineral corticoid receptor, while other steroids act as an agonist.^9^ Furthermore, vamorolone does not translocate to the nucleus, and therefore should not induce side effects that are due to the activation of transcription of genes with an glucocorticoid-response element. In a clinical trial in DMD patients, it was shown that the compound improved the time to rise from the floor after 6 months of treatment compared to placebo.^10^ The clinical trial also suggested similar efficacy to deflazacort and prednisolone.

For the dystrophin-restoring approaches, the majority of marketed treatments for DMD were granted under accelerated approval by the Food and Drug Administration (FDA) in the US, based only on increased expression of a partly functional dystrophin, and functional evidence that disease progression is improved upon treatment is for now lacking.^11^ For the exon skipping compounds, confirmatory trials are ongoing for eteplirsen, casimersen and golodirsen (Table 1). For viltolarsen the primary endpoint (time to rise from floor) was not met after one year of treatment, and an open label stage to capture longer term treatment data is currently ongoing. For the adeno-associated viral vector (AAV) micro-dystrophin gene therapy, delandistrogene moxeparvovec (Elevidys), the primary endpoint was not met: there was no significant difference between the points scored on the north star ambulatory assessment scale between the treated and the placebo group after one year of treatment.^12^ However, for various secondary endpoints, treated patients performed better than the placebo group, including the time to climb 4 stairs, time to walk/run 100 meter and the 95^th^ percentile of the stride velocity (the velocity of the 5% of quickest strides measured by an activity monitor, an endpoint that has been validated by the European Medicines Agency (EMA)).^12^ Elevidys received full marketing authorization for ambulant patients aged 4 and older from the FDA despite missing the primary endpoint.

Finally, in 2014, EMA issued a recommendation for a conditional marketing authorisation based on *post hoc* results of a phase II study showing a beneficial effect on the distance walked in the 6 min walk test for Ataluren compared to placebo.^13^ As this was a *post hoc* exercise, a confirmative study was considered needed. However, the primary endpoint was not met in the pre-specified primary efficacy populations in two confirmatory trials. During the last renewal procedure, the CHMP discussed the observed delay of 3.5 years in the time of loss of ambulation in patients enrolled in the STRIDE registry receiving commercially available treatment with ataluren compared to a group of patients enrolled in a natural history dataset (CINRG-DNH). Nevertheless, the CHMP considered that the interpretability of the results was hampered by the methodological limitations and uncertainties and that these results could not overrule the results of the placebo-controlled phase 3 studies. Accordingly, the CHMP did not recommend the annual renewal of the conditional marketing authorisation for Translarna and the decision was followed by the EC.^14^

## Goal of the meeting

From the introduction above, it is clear that showing clinical benefit for treatments that are approved or in development for DMD has often been challenging. The World Duchenne Organization convened a drug development round table with stakeholders on May 6 2025 in Rome in with in-person and online participants. The goal was to discuss a way forward for clinical trial design, how to cope with the differences in the steroid regimens, aspects around gene therapy and real world data collection.

This meeting consisted of representatives from academia, patient organisations, and regulatory specialists and a data analytics expert. Industry representatives were present as observers. The meeting was held under the Chatham House Rule. To comply with these rules the report summarizes the content of the meeting without disclosing who attended and who said what. While the publication is written by a single individual on behalf of World Duchenne Organisation, speakers and panelists were given and used the opportunity to provide feedback to the manuscript during multiple rounds of editing. Furthermore, the views expressed in this article are the personal views of the author on behalf of meeting participants and may not be understood or quoted as being made on behalf of or reflecting the position of the European Medicines Agency or one of its committees or working parties or any other medicines agency in EU.

## Session 1 clinical trials and challenges of clinical trial design

In order to support a marketing authorisation application, pivotal clinical studies are designed to confirm the findings obtained so far in pre-clinical and other clinical studies. For rare diseases, like DMD, usually only a single pivotal study is done. However, this study then has to be particularly compelling with respect to internal and external validity, clinical relevance, statistical significance, data quality, and internal consistency. As part of internal consistency, regulators will evaluate whether similar effects are demonstrated in different pre-specified sub-populations and whether all-important endpoints show similar findings as outlined in the EMA guidance.^15^

For DMD there are several aspects that make collecting this compelling evidence challenging.

### Challenges of outcome measure selection

First, the disease is heterogeneous. While all patients eventually lose most muscle functions, and loss of different functions happens in the same order in most patients, the age at which each function is lost varies between patients.^16,17^ Secondly, the expected effect of current treatments is a slower disease progression, rather than leading to functional improvement or recovery of lost function. A slower disease progression can only be captured in a cohort of patients who are progressing for a specific clinical outcome measure. For DMD, there is no single optimal clinical outcome measure to capture progression in all patients: as disease progresses, patients lose function, meaning that clinical outcome measures have ceiling and floor effects for different disease stages. Thus, a clinical outcome measure will optimally capture a potential treatment effect in a population for whom the given measure has neither ceiling nor flooring effects.

Furthermore, the outcome measure has to fit with the mechanism of action of the treatment. For instance, the effect of glucocorticoids can be measured relatively quickly in DMD patients for whom these glucocorticoids have an anabolic effect and improve muscle strength, which then also translates into an impact on functional scales such as the North Star Ambulatory Assessment scale (NSAA). By contrast, for approaches that restore the production of partially functional dystrophins, it is known from studies in animal models that a low of dystrophin level will only have a very modest effect on strength.^18^ Thus, longer studies are required to measure a treatment effect in terms of clinical benefit. The same holds for approaches that aim to reduce muscle breakdown.

Therapeutic approaches for DMD target muscle and as such, another aspect that influences the treatment effect is the amount of muscle a patient still has when the intervention starts. Generally speaking, earlier treatment is expected to have a larger and longer therapeutic benefit and pathology starts already in utero.^17^ However, in very young patients, a slower trajectory is difficult to detect as DMD patients at age 4–5 will improve in most motor function scales like the NSAA also in the absence of treatment, because of the neurological development exceeds the impact of muscle breakdown. They will then plateau and thereafter start to decline., but there is also variability in this pattern, i.e., age at which this peak is reached, how high the peak is and when patients start to decline vary across individuals.^16^ In light with the above, the interpretation of the scores in the clinical outcome measures and in particular in composite ones such as NSAA should consider the age / disease stage, i.e., a low score in a young patient could imply that the given motor milestone has not been reached, but it may be attained once the subject develops strength and function further and NSAA scores will then increase, while the same low score in an older patient most likely imply an irreversible loss of function.^19^

Finally, In connection with the amount of muscle that is preserved at the time of the intervention and developmental aspects, gene therapies imposes an additional challenge when given at an early stage, as explained later.

### Learning from past trials

In light of the above, it is good to discuss the placebo-controlled trials that recently failed to meet their primary endpoint in a 12 month clinical trial, i.e., Elevidys,^12^ fordadistrogene movaparvovec (the micro-dystrophin gene therapy developed by Pfizer)(https://www.pfizer.com/news/press-release/press-release-detail/pfizer-provides-update-phase-3-study-investigational-gene) and viltolarsen (https://www.nspharma.com/ns-pharma-shares-preliminary-results-of-viltolarsen-ns-065-ncnp-01-phase-3-clinical-trial-racer53-study/).

The fact that no difference on the primary endpoint was seen between treated and placebo groups implies that effectiveness is not confirmed. However, it does not necessarily mean that the reason is an ineffective intervention. These treatments are expected to slow down disease progression and the inability to measure a treatment effect in a 12-month clinical trial in 4–7 year old patients, could be due to the fact that most of the enrolled subjects were in a stable stage of the disease as measured with the primary endpoint (NSAA for the gene therapies, and rise from supine for viltolarsen).

A duration of 12 months may be too short to detect a slower disease progression. This is further underlined by the fact that for givinostat there was no significant difference between treated and placebo groups measurable after 12 months, while there was at 18 months for their primary endpoint as specified by the applicant.^8^

### Non-ambulant patients

From the patient perspective, it is clear that patients do not want to lose another function. As such, therapeutic options should not only be developed for and evaluated in ambulatory patients. In non-ambulant patients, the maintenance of arm, hand and respiratory/cardiac function is perceived as clinical benefit by patients and caregivers. However, progression for the loss of these functions is slower than that of ambulatory function.^20^ This means that a treatment effect likely will take longer to be able to measure a detectable difference and clinical trials will need to be even longer than in ambulant patients. Additional challenges in this group are that most will have comorbidities such as cardiomyopathy, respiratory failure and osteoporosis.

Furthermore, data from natural history sets including non-ambulant DMD patients are much more limited (see session 4).

### How to optimize trial design

When studying a dossier for marketing authorization, regulators will evaluate whether all-important primary and secondary endpoints show similar findings. However, the ideal DMD cohort to show efficacy in the primary outcome measure, likely will be a suboptimal cohort to show efficacy in other, secondary, outcome measures.

It is therefore recommended to understand the dynamics of the clinical outcome measures in the condition in order to carefully select those with similar dynamics as primary and secondary endpoints. The selection of a study population as proposed above will likely reduce the variability in the trial and may reduce sample size needs or increase the statistical power. However, it is acknowledged that this decision entails a risk of facing difficulties if a broad indication is pursued at the time of the marketing authorization application, e.g., the treatment is only approved for the patients who fulfill the inclusion criteria of the clinical trial. The developers should carefully consider this trade off and carefully plan extrapolation strategies.

It is possible to enroll a large group of subjects and prespecify a primary efficacy population (i.e., those patients in whom the largest treatment effect is expected as per the primary efficacy endpoint). This was done in the givinostat study.

### Placebo vs. external controls

The use of placebo is accepted by the patient community. However, it is a considerable burden, especially now that trials of 18 months and longer are common in the DMD field. Single arm trials using external information from natural history data would be preferred by the patient community. However, from a regulatory perspective, randomised controlled trials are the standard for providing confirmatory evidence on the efficacy of an investigational treatment. The applicant is responsible for justifying the reasons for deviation from the expected standard as outlined in a reflection paper on establishing efficacy based on single-arm trials submitted as pivotal evidence in a marketing authorisation application.^15^ A critical aspect of a trial with a single arm is “treatment effect isolation”, i.e., knowing the observed effect is due to the treatment rather than being part of natural heterogeneity. Only in exceptional cases can this be perfectly achieved: when the observed individual outcomes for the planned endpoint could not occur without effective treatment within the designated follow-up period for any trial participant. As an example, the marketing authorisation application for Zolgensma as a treatment of spinal muscular atrophy has a single arm trial as pivotal evidence showing that the survival and motor milestones achieved in the study population largely exceed the expectations of the natural history of spinal muscular atrophy type 1.^21^

However, for a disease like DMD, where the treatment effect will be a slower progression, external data is needed for a comparison and it will be very challenging to obtain compelling evidence when comparing to natural history. Ultimately, the regulators need to determine whether causal conclusions can be drawn with sufficient certainty from the single arm trial on the effect of the treatment.

Similarly, there has been a call for better outcome measures, trial design and statistical analyses to allow measuring a treatment effect DMD patients. This need has arisen from the fact that current treatments only have a minor effect on disease trajectory. We could argue that we need better treatments. However, one has to realize that DMD is a disease that affects the whole musculature from birth. Even if we would restore fully functional dystrophin at normal levels in a four-year-old DMD patient, he would not be normal, as he will have accumulated loss of muscle tissue from in utero until the time of intervention. Thus, the nature of the disease is such that demonstrating efficacy may still be challenging, even in a scenario where more efficacious treatments could be developed.

## Session 2 the impact of variability in steroid regimen

While the use of glucocorticoids is a key aspect of the standard of care,^4^ there is no consensus on the therapeutic strategy that maximizes efficacy while minimizing the risks of adverse events inherent to the chronic use. Accordingly, there is a large variability in the age at which glucocorticoids are initiated, the regimen (e.g., daily, every other day, weekend-free, weekend only), type of glucocorticoid (e.g., deflazacort, prednisone, vamorolone) and weight – adjusted doses. These variations in practice can be due to country-specific standards, efforts to manage the adverse effects of chronic glucocorticoid use, and fear of glucocorticoids by both physicians and patient families. As such, efforts to require a strict and consistent glucocorticoid regimen that aligns with what is recommended in the standards of care for all participants entering a trial can face resistance from healthcare providers and families. Acknowledging this lack of consensus, eligibility criteria often require the patient to be under a stable dose of glucocorticoids for a certain time before enrollment (usually 6 months) and remain under same treatment while on trial, but do not specify dose or regimen. Furthermore, trials will often stratify for the glucocorticoid regimen (daily vs on/off regimen). Still, there will be a lot of individual variation, especially as there are also patients where the use of glucocorticoids is initiated to comply with the inclusion criteria.

The age at onset is of particular relevance due to the implication in the cumulative dose and recognized impact on disease trajectory.^22^ For instance two ten-year-old patients may be on the same dose regimen of glucocorticoids, but if one started at age 4, while the other started at age 9.5, the cumulative dose will differ a lot. It is known that disease trajectory depends on the peak function achieved and glucocorticoids can improve the peak function. When glucocorticoids are started at age 7 or later, there will still be an impact on disease trajectory, but the window of opportunity to have an impact on increased peak function is past.^22^ This is an important source of variability that is currently often not accounted for in clinical trials.

The variations in glucocorticoid regimen and exposure will make it more difficult to identify treatment effects, especially when these are small When stratifying, the most pertinent strata to consider are regimen (daily or non-daily) and initiation of steroids (before or after 6 years) as these aspects have the largest impact on disease progression.

The type, dose and regimen and cumulative exposure of glucocorticoids is captured in most natural history datasets, so it is possible account for these variables when using natural history datasets as external controls.

For the non-ambulant patients the variability between patients will be even larger. Most patients nowadays continue using glucocorticoids once ambulation is lost, as this is protective for heart and respiratory function.^23^ However, previously, it was more common to stop glucocorticoids once ambulation was lost.

In summary, it is clear that variability in initiation age has considerable impact on patient trajectories. In future trials, stratification could be considered on glucocorticoid regimen (daily vs. others) and age of initiation (before/after 6 years).

## Session 3. Gene therapy

As mentioned, two gene therapy products have been tested in placebo-controlled trials: Sarepta's Elevidys and Pfizer's fordadistrogene movaparvovec. Both trials used the NSAA as a primary endpoint after 1 year of treatment, both included 4–7 year old patients, both showed micro-dystrophin expression in muscle biopsies of treated patients and both missed their primary endpoint for motor function improvement^12^ (https://www.pfizer.com/news/press-release/press-release-detail/pfizer-provides-update-phase-3-study-investigational-gene). For fordadistrogene movaparvovec, secondary endpoints did not differ between treated and placebo patients and Pfizer stopped the development of this gene therapy. By contrast, for Elevidys some of the secondary endpoints showed a trend towards improvement for treated patients.^12^ Elevidys received full approval from the FDA for DMD patients in June 2024 and accelerated approval for non-ambulant patients, after the CBER director overruled the negative opinion of the reviewing committee.

Physicians involved in clinical trials with gene therapy see noticeable improvement in treated patients, e.g., patients can do tasks like rising from the floor or the 10 meter run, faster. However, these changes are currently not captured in the NSAA score, which only reflects whether patients are able to do the task, and not how fast they perform and whether this is an improvement compared to baseline (Table 2).

The gene therapy approach is not without adverse effects in DMD and severe adverse events have been reported, including liver failure for adeno-associated viral vector 74 (AAV74) (lethal in one elevidys-treated patient at the time of the meeting, with an additional death reported at the time of writing this report (https://www.reuters.com/business/healthcare-pharmaceuticals/sarepta-reports-second-case-liver-failure-death-after-its-gene-therapy-treatment-2025-06-15/)). For fordadistrogene movaparvovec thromobotic microangiopathy has been reported, which likely is an AAV9 specific side effect. Myositis and myocarditis have been reported for both AAV74 and AAV9, due to an inflammatory response to the AAV capsids. Administration of AAV vectors make patients ineligible for treatment with future AAV-based gene therapy.^24^

A subset of patients is at risk of developing an adaptive immune-mediated response to the micro-dystrophin. This has occurred in various gene therapy drug development programs in DMD.^25^ However, the region triggering the immune response (hinge region 1 and flanking parts) has been identified and patients carrying deletions encoding this domain, and thus at high risk for developing this immune response, are excluded from trials and a contraindication has been included in the labelling.

Note that during the writing of the report, following the death of two non-ambulant DMD patients from acute liver failure after receiving Elevidys, trials testing Elevidys in non-ambulant patients were paused and distribution for treatment of non-ambulant patients suspended. Furthermore, during the writing of the manuscript the CHMP gave a negative opinion for Elevidys for the treatment of 3–7 year old ambulant DMD patients (https://www.ema.europa.eu/en/medicines/human/EPAR/elevidys).

### Risks and uncertainty

As Elevidys was approved by the FDA without ascertaining clinical benefit, there is uncertainty with physicians and patients. They are asked to make a decision on whether to undergo this treatment, knowing the risks, knowing that treatment can happen only once, but without knowing if and how much benefit there is. At the same time there is also the fear of missing out: if there is a treatment effect, it is likely larger when the treatment is given earlier, so waiting for additional, longer term data to come in, might take away the window to attain optimal benefit. Having said this, it is also possible that treating at a very young age will be suboptimal. AAV vectors do not integrate, so with muscle growth, vectors and thus micro-dystrophin levels will be diluted. Notably, muscle mass increases about 20-fold from newborn to adulthood. Furthermore, as the micro-dystrophin is not fully functional, there will be loss of protein levels with time due to muscle turnover. For now, there is little evidence on how long micro-dystrophin will persist, as muscle biopsies are only done within a clinical trial setting and not afterwards. Thus, the optimal timing for the treatment is currently unknown. Efforts are ongoing to improve AAV and gene therapy technology, and as retreatment is currently not possible, there is also the fear of missing out on a future, more optimal treatment.

For older patients there are other uncertainties: their cardiac and skeletal muscle tissues will be in a hyperinflammatory state due to chronic pathology, which may heighten immunological responses and thus increase the risk of severe side effects. Furthermore, for older patients, cardiac and respiratory reserve is impaired and they may not be able to cope with the adverse effects and this may result in very severe adverse effects and even death (https://www.reuters.com/business/healthcare-pharmaceuticals/sarepta-reports-second-case-liver-failure-death-after-its-gene-therapy-treatment-2025-06-15/). In clinical trials patients with a left ventricular ejection fraction less than 40% are excluded from participation However, this was not included as contraindication on the label, so patients with a low ejection fraction are eligible receiving treatment in the commercial setting, despite risking severe adverse effects.

As currently over 800 patients have been treated with Elevidys within trials or in a post-marketing setting, and some patients were treated many years ago, there are now also DMD patients dying with gene therapy due to other causes. There is currently no standardized procedure to do autopsy in these individuals. While acknowledging the sensitivity of this topic, being able to do an autopsy would provide important information on the longevity of the transgene, the biodistribution over different skeletal muscles and also the transduction of the heart. During the consenting process for gene therapy there are many discussions about what will happen when severe adverse effects occur. However, the possibility of doing an autopsy later in time is not discussed.

### Improved trial design for assessing gene therapy efficacy

In hindsight, it is clear that the cohort selected for the placebo-controlled trial was suboptimal, as in 4–7 year old DMD patients there is a lot of variability in the NSAA trajectory: some will improve, some will be stable and some will decline.^19^ In addition, it is now known there is an impact of the increased glucocorticoid dose given just prior to the gene therapy treatment, which has a positive impact on motor function that can persist for months after weaning patients off the higher dose.

After age 7, the trajectories of DMD patients are less variable and they mostly decline.^19^ When having to redo the placebo-controlled trials, including patients over 7 and a using a more nuanced NSAA, to also allow picking up minor improvements, such as doing specific tasks with more ease or quicker, might be able to tease out treatment effects.

New outcome measures are continuously developed, but their validation takes time. Furthermore, collecting the natural history dataset for these measures takes time. While timed function tests allow picking up that patients are doing a task faster, the challenge is that sometimes they are difficult to interpret with regards to clinical meaningfulness.

In summary, there are many unknowns and uncertainties yet related with gene therapy. Prospective data collection on patients treated with gene therapy during life and post-mortem will allow the field to increase knowledge on these aspects.

## Session 4. Real world evidence

Real-world data has been recognized as a potentially valuable source of data to support regulatory decision-making on medicines. Real-world data may provide valuable information to optimize clinical trial design in the pre-authorisation phase. As an example, real world evidence has allowed the identification of prognostic factors affecting disease trajectories for DMD patients measured by specific clinical outcome measures, which is useful information to identify the appropriate study population for clinical trial inclusion.^26^ Real world data may also be useful to contextualise post-authorisation clinical studies aiming to inform about long-term effectiveness and safety or support an extension of the indication in populations not included in the indication at the time of the marketing authorisation application.

Among all potential uses of real world data to support regulatory decision-making on medicines, the discussion in the meeting focused on its use as a comparator for a single arm trial to collect pivotal confirmatory evidence for a marketing authorisation application or to support the extension of the indication/label. Indeed, a critical feature of single arm trials is that one does not know the trajectories of the subjects in the trial had they not been treated, which makes it challenging to evaluate the causal effect of the potential treatment on the observed outcome. The aforementioned reflection paper^15^ on use of real-world data in non-interventional studies to generate real-world evidence for regulatory purposes provides additional insights about real world data, including its use in studies with aiming to assess whether a treatment leads to clinical benefit.^27^

Beyond the considerations discussed in the aforementioned concept papers, in the specific context of use in DMD, the following aspects were highlighted during the discussion:

First, the panel discussed aspects revolving around what is been collected (quantity aspects). It was agreed that there is a paucity of real-world data for the older and non-ambulant patients, a population for which the unmet medical need is the largest. Second, it was discussed that a big challenge in the real-world data collection is that you have to be a fortune teller: you have to prospectively collect data for future use and it is not always clear which aspects / data will be critical at the time the data will be used. For example, EMA has issued a qualification opinion for stride velocity 95th centile as primary endpoint in studies in ambulatory DMD studies,^28^ but it is unclear to what extent these data are now prospectively collected in the real-world datasets. Should a new clinical trial use this endpoint, but the information about the endpoint not be available in real-world datasets, a comparison between single arm trials and real world data groups regarding this endpoint is not possible.

The panel also discussed aspects revolving about how data is being collected (quality aspects). It seems that clinical outcome measures may have not been systematically collected during the entire data collection period of a registry.

Further, it was highlighted that the definition of certain disease milestones may vary across datasets, which represents a big challenge for setting that milestone as a clinical outcome. Additionally, the impact of missing data was acknowledged. Usually real world data are collected as part of clinical care visits. While missing data is not expected to have an impact on the quality of the clinical care, it negatively impacts the potential usability of the dataset as external information. Furthermore, the panel acknowledged that the care for DMD patients continuously improves, even more now with approved treatments, and with it natural history trajectories will change. This is a challenge not in connection with the data collection but with the data analysis. In essence, the potential impact of the improvement in the standard of care needs to be accounted in the statistical analysis plan.

DMD families have high expectations of the use of real world data and its potential to generate real world evidence to support decision making process. However, challenges around the data collection and data analyses are recognized and the reality is that not all real world datasets can be used.

Finally, it was discussed that real world evidence data are often captured in silos, without alignment of what is being captured. Towards the future it will be important to define what is captured, how it is captured and at which frequency. In addition, identifying who has already collected what is important to connect data and to move forward in a coordinated way. Both the quality and the quantity of the data are important, but collecting high quality in high quantity will require significant investment.

In summary, it is clear that real world data have a place in clinical research and can support regulatory decision-making on medicines for DMD, but this will require alignment of the field to continuously collect high quality and quantity data. Furthermore, the methodological aspects highlighted in the reflection papers should be carefully considered to be able to conclude on causal effects.

## Learnings from the meeting

The goal of the meeting was to raise awareness across stakeholders of the challenges facing drug development for DMD and to have a constructive discussion about how to plan for the future. This mission was achieved. Below the most important learnings for the participants are listed:

- Short trials are often not possible for DMD and there is no one size fits all trial design that will work for all treatment approaches, or one outcome measure that applies to all disease stages. While it is clear DMD patients and caregivers prefer placebo-controlled trials to be short, it is even more important to them that clinical trials are appropriately designed and conducted so results allow to conclude on causal effects on efficacy and safety.
- The biology and pathology of DMD is such that detecting a treatment effect for DMD for treatments currently in development will be likely challenging.
- The peak function determines the disease trajectory and thus has an impact on outcome measures; variation in care (e.g., when glucocorticoids are initiated or different glucocorticoid regimen) has an impact on peak function.
- The use of glucocorticoids could be considered as a stratification factor. It is impossible to account for the 20 + different glucocorticoid regimen, but it is feasible to use the 2 most important aspects: continuous or on/off regimen and time of glucocorticoid initiation before or after age 6, as these influence the peak function to the largest extent.
- For gene therapy there are many unknowns as yet: when is the optimal time for treatment? How to optimally manage adverse effects? What is the long term effectiveness?
- The use of real-world data for regulatory purposes requires the collection of high quality data. Data collection should take into account the most recent developments in the field including not only medicinal products but also clinical outcome measurements and endpoints. Overall, the field needs to break silos and agree as much as possible in the data collection strategies (what to collect, how to collect and at which frequency) and have a data dictionary for definitions. The community should increase efforts to collect real world data in older and non-ambulatory subjects as this population represents the biggest unmet medical need and the data is currently scarce.
